# Human cytomegalovirus UL76 induces chromosome aberrations

**DOI:** 10.1186/1423-0127-16-107

**Published:** 2009-11-25

**Authors:** Voon-Kwan Siew, Chang-Yih Duh, Shang-Kwei Wang

**Affiliations:** 1 Department of Microbiology, Institute of Medicine, College of Medicine, Kaohsiung Medical University, 100 Shih-Chuan 1st Road, Kaohsiung 80708, Taiwan; 2 Asia-Pacific Ocean Research Center, National Sun Yat-sen University, 70 Lien-Hai Road, Kaohsiung 80424, Taiwan

## Abstract

**Background:**

Human cytomegalovirus (HCMV) is known to induce chromosome aberrations in infected cells, which can lead to congenital abnormalities in infected fetuses. HCMV UL76 belongs to a conserved protein family from herpesviruses. Some reported roles among UL76 family members include involvement in virulence determination, lytic replication, reactivation of latent virus, modulation of gene expression, induction of apoptosis, and perturbation of cell cycle progression, as well as potential nuclease activity. Previously, we have shown that stable expression of UL76 inhibits HCMV replication in glioblastoma cells.

**Methods:**

To examine chromosomal integrity and the DNA damage signal γ-H2AX in cells constitutively expressing UL76, immunofluorescent cell staining and Western blotting were performed. The comet assay was employed to assess DNA breaks in cells transiently expressing UL76.

**Results:**

We report that stably transfected cells expressing UL76 developed chromosome aberrations including micronuclei and misaligned chromosomes, lagging and bridging. In mitotic cells expressing UL76, aberrant spindles were increased compared to control cells. However, cells with supernumerary centrosomes were marginally increased in UL76-expressing cells relative to control cells. We further demonstrated that UL76-expressing cells activated the DNA damage signal γ-H2AX and caused foci formation in nuclei. In addition, the number of cells with DNA breaks increased in proportion to UL76 protein levels.

**Conclusion:**

Our findings suggest that the virus-associated protein UL76 induces DNA damage and the accumulation of chromosome aberrations.

## Background

Myriad chromosomal or genomic abnormalities are common in viral lytic and latent infected cells, and even in virus-associated tumors. Recent studies have consistently shown that cellular defense mechanisms recognize infections involving a wide range of DNA and RNA viruses as abnormally damaged DNA, including human immunodeficiency virus (HIV), Epstein-Barr virus (EBV), herpes simplex virus (HSV-1), adenovirus, and Simian virus 40 (SV40). DNA damage responses and repair pathways are thus activated after infection [1-5]. To counteract these intrinsic cellular defenses, the viruses have evolved strategies to mitigate DNA damage signal transduction, attenuate DNA repair pathways, and modulate cell cycle progression [6-11]. Overall, cells infected with virus accumulate DNA damage that is directly linked to viral pathogenicity and presumably leads to genomic instability.

HCMV is a ubiquitous pathogen in humans, and following primary infection sustains an asymptomatic latent infection. During life-long infection, the viral life cycle displays multiple phases within the human body. These include active lytic replication, a low level of persistent infection, and insidious latency. Notable clinical complications associated with HCMV infection are in utero congenital infection, opportunistic infection in immunocompromised patients, cardiovascular diseases, and possible malignant tumors [12-15].

Evidence indicates that HCMV causes chromosome aberrations following infection. These abnormalities include selective chromosome breakages, chromosome pulverization, premature chromatid condensation, and centrosome structural injury [16-19]. Specifically, UV-inactivated HCMV is capable of inducing site-specific breaks at positions 1q42 and 1q21 on chromosome 1 and centrosome injury, indicating that the damage is related to virion-associated proteins and unrelated to de novo viral protein production [16,20,21]. These specific DNA break-points may explain the congenital hearing loss of HCMV-infected neonates.

HCMV *UL76 *encodes a protein belonging to the conserved UL24 protein family from herpesviruses [22]. Several lines of evidence have shown that the UL76 protein and its family members govern multiple functions. During a typical lytic replication cycle, UL76 transcripts are expressed with true-late kinetics [23]. The UL76 protein predominantly localizes to the nucleus and nucleolus, resulting in a significant reduction in the number of promyelocytic leukemia (PML) bodies [24,25], where HCMV gene expression and genome replication initiates [26]. Functional analyses of HCMV coding contents were performed by Tn-mediated insertion, and a recombinant virus with an insertion in *UL76 *resulted in a significant reduction in virus production [27]. Similarly, deletion of the entire *UL76 *ORF resulted in a total loss of virus production [28,29]. We previously demonstrated that UL76 is able to regulate both repression and activation of gene expression [24]. Particularly, UL76 is capable of repressing the expression of replication-essential genes in a dose-dependent manner, including UL54 (DNA polymerase), UL123 (major immediate-early gene) and UL112 (major early gene, pre-replication factor) [24]. In addition, UL76 is involved in the late stage of egress, and it is present in three types of mature viral particles, the virion, NIEP, and DB [23]. Virus-associated UL76 presumably plays a role in the modulation of gene expression once delivered into the cell at a very early stage of viral infection. This speculation is partly based upon the facts that there are significant decreases in protein production in UL76-expressing cells for IE (IE1p72 and IE2p82) proteins, early gene products UL44 (DNA polymerase processivity factor) and UL57 (single-stranded DNA binding protein), and the late gene encoding UL99 (tegument protein) [23]. Consistent with the repression of gene expression, HCMV production is dramatically inhibited in UL76-expressing cells. In addition, in an HCMV genome-wide expression assay, UL76 is over-expressed in hematopoietic CD34^+ ^cells latently infected with HCMV [30,31]. Taken together, these results suggest that *UL76 *is not only an essential gene for lytic replication but also implicate *UL76 *in viral latency.

During the course of this study, Knizewski and colleagues proposed that the UL76 protein family contains a potential endonuclease motif (Pfam accession number: PF01646) using computational analysis [32]. We show here that in UL76-expressing cell lines chromosome aberrations, micronuclei and chromosomal misalignments (laggings and bridgings) were significantly increased. Further, an increased number of these cells exhibited enhanced nuclear foci containing phosphorylated histone γ-H2AX. We also show that UL76 induces DNA breaks in proportion to its protein levels, and marginally subverts mitotic fidelity by inducing aberrant spindles and supernumerary centrosomes relative to control cells. Our results therefore suggest that HCMV UL76 may be a source of chromosomal abnormalities, and the fundamental alteration of the cellular biochemical environment may modulate viral production.

## Methods

### Cell cultures

Human embryonic lung cells (HEL299, ATCC), COS-1 cells, and human glioblastoma U-373 MG cells were maintained in Eagle's MEM supplemented with 10% fetal bovine serum (FBS). Methods for the construction, selection and maintenance of G418-resistant cells expressing UL76 were published previously [23]. Stably transfected cell lines expressing UL76 were designated S1, S3, S4, and S5, and the parallel control cell line stably transfected with the cloning vector pBK-CMV was designated P7. These stable cells were routinely maintained in the presence of 25 μg/ml G418 (Pierce Biotechnology Inc., Rockford, IL, USA).

### Antibodies

Primary mouse monoclonal antibodies (mAbs) used in this study include anti-α-tubulin (clone B-5-1-2, Sigma-Aldrich, St. Louis, Mo, USA), anti-γ-tubulin (clone GTU-88, Sigma-Aldrich, St. Louis, MO, USA), anti-γ-H2AX (Ser139) (clone JBW301, Upstate Biotechnology, Charlottesville, VA, USA) and anti-myc (Invitrogen, Carlsbad, CA, USA).

### Plasmid construction and transient protein expression

A 975-bp DNA fragment encompassing nucleotides 111 258 to 112 232 of HCMV AD169 (annotated genome accession number NC001347) encoding full-length *UL76 *was amplified by polymerase chain reaction using the 5' and 3' primers, **GGATCC**CACCATGCCGTCCGGGCGT and **GAATTC**CTAAAGACCGTGTGGGACGGCA, respectively. BamHI and EcoRI (in bold) sites were generated at the ends of each amplified DNA fragment. The cloning vector pEF1/Myc-His (Invitrogen, Carlsbad, CA, USA) and the amplified UL76 DNA were digested with BamHI and EcoRI and re-ligated. The resulting plasmid was designated pUL76-myc and encoded a myc epitope at the C terminus of *UL76*. Transient expression of UL76 was achieved by seeding 2 × 10^5 ^cells in a 6-well culture dish. Plasmid DNA was transfected with Lipofectamine Plus reagent (Invitrogen, Carlsbad, CA, USA). Total DNA for each transfection was maintained at a constant 1 μg per well by addition of the empty cloning vector pEF1/Myc-His where necessary.

### Indirect immunofluorescent analyses

Detailed protocols for immunofluorescent cell staining have been described [23]. In brief, stably transfected U-373 MG cells were seeded onto a coverslip (20 × 10^4 ^cells per well) in six-well culture plates one day before staining. The following day, the cells were fixed in 2% paraformaldehyde in phosphate-buffered saline (PBS) for 10 minutes at room temperature and then permeabilized with 1% Triton X-100 in PBS for 20 minutes at 65°C. To detect mitotic spindles, cells were stained with α-tubulin or γ-tubulin monoclonal antibodies at a dilution of 1: 500 and incubated for 30 minutes at 37°C in a humidity chamber. After extensive washing in PBS, the cells were immersed in a solution containing one μg/ml DAPI and the secondary antibody Texas Red-conjugated goat anti-mouse immunoglobulin G (1: 1000 dilution, Vector Laboratories, Burlingame, CA, USA) for 30 minutes at 37°C. Characteristics of chromosome aberrations including the presence of micronuclei, abnormal spindles, chromosomal misalignments, chromosomal laggings and mitotic bridgings were recorded. To assess the induction of the γ-H2AX protein and the formation of foci by HCMV UL76, the γ-H2AX foci were visualized by immunofluorescent staining. Asynchronous stably-transfected cells were cultured on coverslips and washed twice with PBS, fixed for 10 minutes with 1% paraformaldehyde in PBS, permeabilized with 0.1% NP-40 in PBS for 30 minutes on ice, and then incubated with γ-H2AX mAb (1:1000), followed by goat anti-mouse IgG secondary antibody conjugated to Alexa Flour^® ^488 (Molecular Probe, Eugene, OR, USA). Coverslips were air dried and preserved in Prolong^® ^Gold anti-fade reagent (Molecular Probe, Eugene, OR, USA). Confocal images were acquired with a laser scanning confocal microscope (Olympus FV1000). Images were processed with Adobe Photoshop (version 9.0) software.

### Western blot analyses

γ-H2AX (Ser139) was detected in cells following a protocol for acid extraction of protein. Asynchronous cells were cultured to 90% confluency in 10 cm dishes and harvested by centrifugation. Cell pellets were suspended in lysis buffer containing 10 mM HEPES, pH 7.9, 1.5 mM MgCl_2_, 10 mM KCl, 0.5 mM DTT, and 1.5 mM PMSF. HCl was added to a final concentration of 0.2 M and the acidified protein extracts were incubated for 30 minutes on ice. Acid soluble proteins were dialyzed in 0.1 M acetic acid with several changes of ddH_2_O. To examine the production of UL76 or α-tubulin by Western blotting, transfected cells were lysed in RIPA buffer (50 mM Tris pH 7.5, 150 mM NaCl, 1% NP-40, 0.05% sodium deoxycholate, and 0.01% SDS) containing complete protease inhibitor cocktail (Roche, Mannheim, Germany). Soluble proteins were collected and total protein was quantified using a Bio-Rad Bradford protein assay kit (Bio-Rad, Hercules, CA, USA). Thirty micrograms of protein were boiled for 5 minutes in 5% β-mercaptoethanol reducing Laemmli sample buffer, and proteins were separated by SDS-10% PAGE then transferred to PVDF membrane (Hybond-P, GE healthcare, Piscataway, NJ, USA) in a Towbin transfer buffer (48 mM Tris, 39 mM glycine [pH 9.2]). Membranes were blocked in Tris-buffered saline (TBS, 50 mM Tris, 150 mM NaCl [pH7.5]) containing 1% skim milk for one hour and then probed with the anti-myc or anti-α-tubulin antibody indicated in the text followed by a 1:30,000 dilution of horseradish peroxidase-conjugated anti-mouse immunoglobulin G (GE Healthcare, Piscataway, NJ, USA). Chemiluminescent signals were generated using Lumi-Light^Plus ^Western blotting substrate (Roche, Mannheim, Germany) and recorded on Hyperfilm™ ECL (GE Healthcare, Piscataway, NJ, USA).

### Comet assay

To assess induction of DNA breaks by HCMV UL76 in vivo, pUL76-myc was transiently expressed in HEL299 and COS-1 cells. One day post-transfection, DNA breaks were detected using the CometAssay kit (Trevigen, Gaithersburg, MD, USA). Cells were harvested and combined to a concentration of 1 × 10^5 ^cells/ml in molten low-melting agarose at a ratio of 1:10 to immobilize the cells onto the CometSlide. Following a gentle lysis in 1% sodium lauryl sarcosinate cells were treated with alkaline solution (0.3 M NaOH, 1 mM EDTA) for one hour in the dark to unwind, denature the DNA and hydrolyze the sites of damage. Cells were then subjected to alkaline electrophoresis at 1.0 volt cm^-1 ^at 4°C for one hour. Slides were subsequently rinsed by dipping several times in ddH_2_O and then immersed for five minutes in 70% ethanol. Slides were allowed to air dry and cells were visualized by staining with SYBR Green and scored by epifluorescence microscopy for the distribution of DNA between the "tail" and the "head". At least 75 randomly selected cells were scored per sample.

### Statistical analyses

Statistical significance was assessed using the chi-square two-tailed test for independent samples. At least three independent experiments were evaluated for each cell population and the mean ± standard deviation is given.

## Results

### HCMV UL76-expressing cells induce micronuclei formation

**Figure 1:**
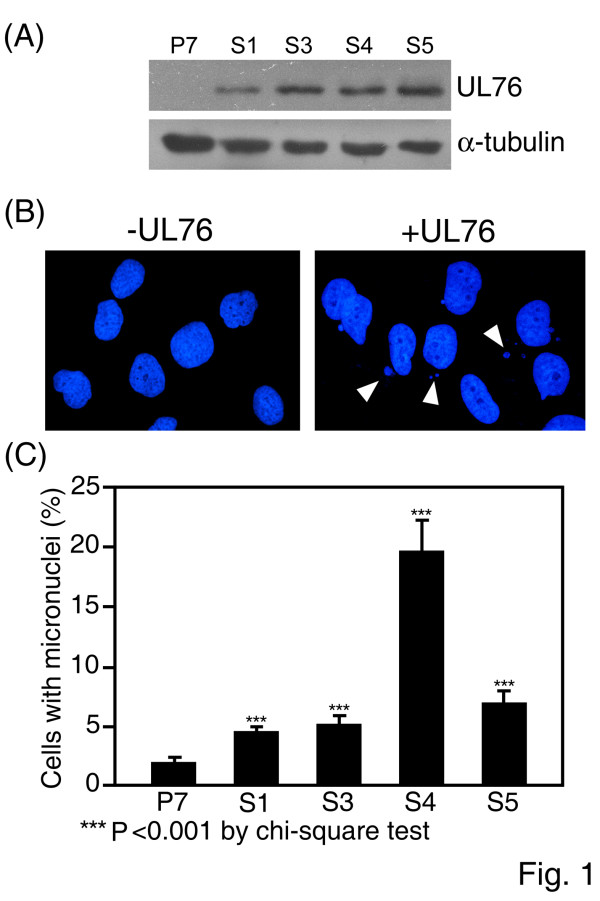
**Induction of micronuclei in HCMV UL76-expressing human glioblastoma (U-373 MG) cells**. (A) Western blot analysis of UL76 protein in cells. α-tubulin was used as a loading control. (B) Representative images of micronuclei (arrow) detected by DAPI staining of U-373 MG cells stably transfected with control vector (-UL76), or with a plasmid expressing UL76 (+UL76). (C) Quantification of micronuclei in an asynchronous cell population expressing either vector (P7) or UL76 (S1, S3, S4, and S5). The average data point was calculated from three independent experiments. At least 4000 cells were counted for each cell line.

We previously established human glioblastoma cells stably expressing UL76 by transfection of pUL76-CMV (23). S1, S3, S4, and S5 are UL76-expressing cells, and P7 is transfected with the cloning vector pBK-CMV (Fig. 1A). To examine the cellular effects upon UL76 expression, nuclear morphology was first examined by staining asynchronous stable cells with DAPI. Surprisingly, we observed that UL76-expressing cells developed micronuclei, a sign of chromosome aberration (Fig. 1B). S1, S3, S4, and S5 showed higher percentages of micronuclei formation induction than P7 control cells. The percentages and chi-square values of micronuclei in UL76-expressing cells compared to control P7 cells (2.3%) were as follows (Fig. 1C): S1 (5.1%; χ^2 ^= 34.74; P < 0.001), S3 (5.8%; χ^2 ^= 52.58; P < 0.001), S4 (21.6%; χ^2 ^= 556.88; P < 0.001), and S5 (7.7%; χ^2 ^= 109.69; P < 0.001). These results indicated that chromosomal damage accumulated in UL76-expressing cells. Therefore, it is plausible that DNA damage response signals, cell cycle checkpoint surveillance and DNA repair machinery do not function normally in cells expressing UL76. This finding prompted us to examine chromosomal alignments in mitotic cells, in which abnormalities may increase micronuclei formation in resting cells.

### UL76-expressing cells induce chromosomal misalignments during mitosis

**Figure 2:**
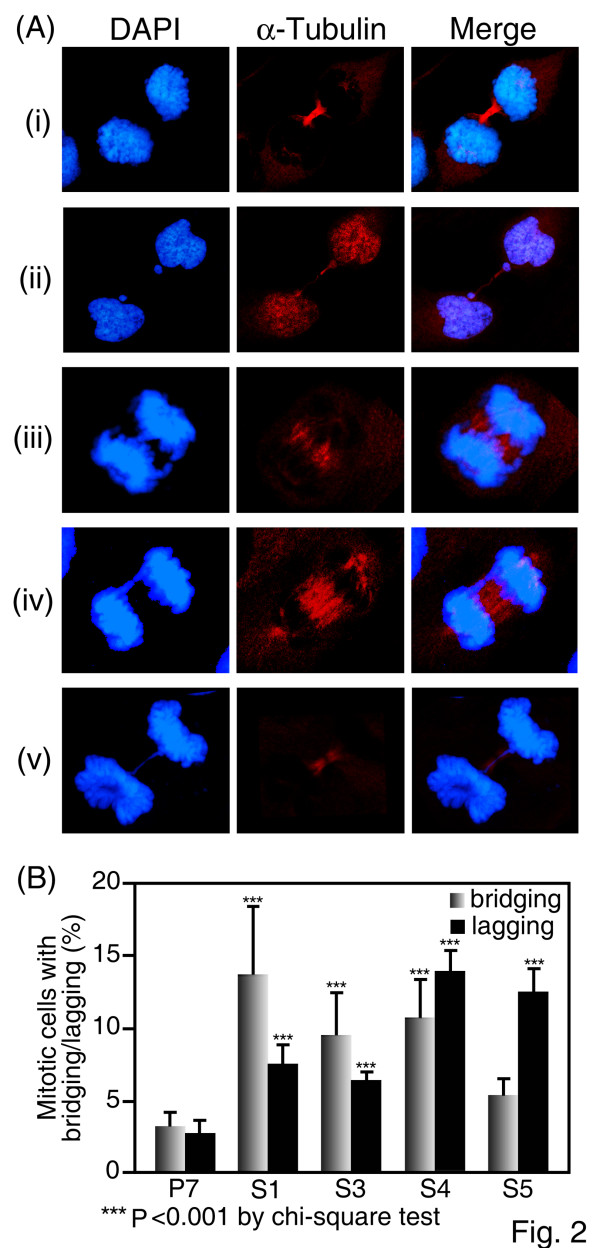
**Induction of chromosomal misalignments in the HCMV UL76-expressing glioblastoma cells (U-373 MG)**. (A) Representative images of chromosomal misalignments induced in cells stably expressing HCMV UL76; chromosomal laggings (ii and iii), and mitotic bridges (iii to v) in glioblastoma cells constitutively expressing HCMV UL76. In parallel control experiments, empty vector-transfected cells were used as normal mitotic cells (i). Chromosomes were stained with DAPI (blue) and mitotic cells were visualized by immunofluorescent staining using a monoclonal antibody to α-tubulin (red). Two side-by-side panels of single-labeled immunofluorescent images and a third panel with an overlapping image are shown. (B) Quantification of chromosomal misalignments in mitotic cells stably expressing HCMV UL76 (S1, S3, S4, and S5) or control cells (P7). The average data point was calculated from three independent experiments. At least 800 mitotic cells were counted for each cell line.

The emergence of micronuclei is related to the mitotic phase of chromosome condensation and segregation. To examine the microtubule spindle and chromosomal aligning pattern at the mitotic stage, UL76-expressing cells and control cells were stained with α-tubulin and DAPI (Fig. 2A). During anaphase, condensed chromosomes are segregated to two poles (Fig. 2A(i)). Mitotic cells with lagging chromosomes show a piece of detached DNA (Fig. 2A(ii)), and bridging chromosomes are characterized by broken chromosomal connections between two segregated chromosomes (Fig. 2A(iii), (iv) and 2A(v)). Mitotic cells with misaligned, lagging and bridging chromosomes were counted (Fig. 2B). The percentages and chi-square values of chromosome bridging in mitotic cells compared to control P7 cells (3.2%) were as follows (Fig. 2B): S1 (13.7%; χ^2 ^= 36.63; P < 0.001), S3 (9.5%; χ^2 ^= 26.16; P < 0.001), S4 (10.7%; χ^2 ^= 27.67; P < 0.001), and S5 (5.4%; χ^2 ^= 2.67, P = 0.077). Percentages and chi-square values of chromosomal lagging in mitotic cells compared to control P7 cells (2.8%) were as follows: S1 (7.5%; χ^2 ^= 15.65; P < 0.001), S3 (6.4%; χ^2 ^= 12.57; P = 0.001), S4 (13.9%; χ^2 ^= 67.24; P < 0.001), and S5 (12.5%; χ^2 ^= 42.08; P < 0.001). The presence of chromosome lagging and bridging in UL76-expressing cells compared to control cells was statistically significant. These findings suggest that UL76 is able to attenuate mitotic checkpoint surveillance and allow mitosis to proceed despite notable chromosome defects.

### UL76-expressing cells moderately enhance aberrations in mitotic spindles and centrosomes

**Figure 3:**
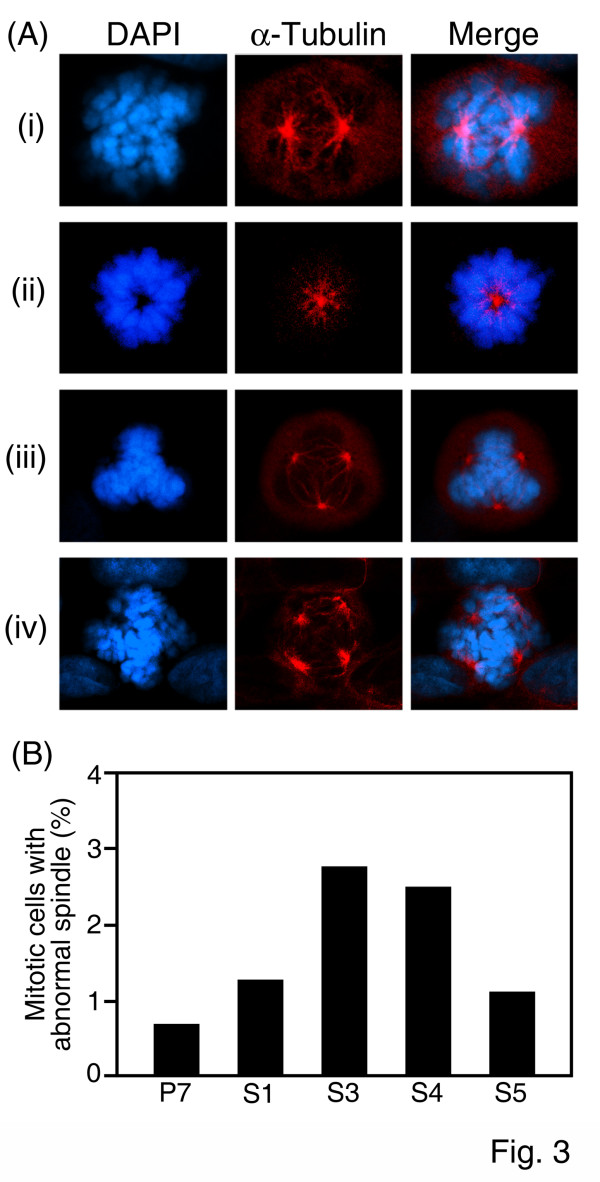
**Emergence of abnormal spindle networks in cells constitutively expressing HCMV UL76**. (A) Representative images of mitotic spindles in empty vector-transfected and UL76-expressing cells, within which normal mitotic bipolar spindles (i) and abnormal monopolar, tripolar and tetrapolar spindles are evident (ii to iv), respectively. The mitotic spindle networks were visualized by using immunofluorescent staining of α-tubulin (red) and the chromosomal alignments were visualized by staining with DAPI (blue). Two side-by-side panels of single labeled immunofluorescent images and a third panel with an overlapping image are shown. (B) Quantification of mitotic spindles in cells stably expressing HCMV UL76 (S1, S3, S4, and S5) and control vector (P7).

In addition to the chromosomal abnormalities, we speculated that the mitotic spindle network and centrosome would be affected by UL76 expression. The integrity of the spindle network and centrosomes were examined and enumerated during the mitotic phase. During mitosis, each cell has a two spindle network (Fig. 3A(i)). In contrast, mitotic cells containing one or more than two spindles are considered abnormal. The images shown in Fig. 3A(ii), (iii), and 3A(iv) reveal mono-, tri-, and tetra-spindle formation during the mitotic stage. The results of this analysis revealed a moderate increase in the percentage of UL76-expressing cells with increased spindle networks compared to P7 control cells [P7 (0.7%), S1 (1.3%), S3 (2.8%), S4 (2.5%), and S5 (1.1%)], but these differences were not statistically significant (Fig. 3B).

**Figure 4:**
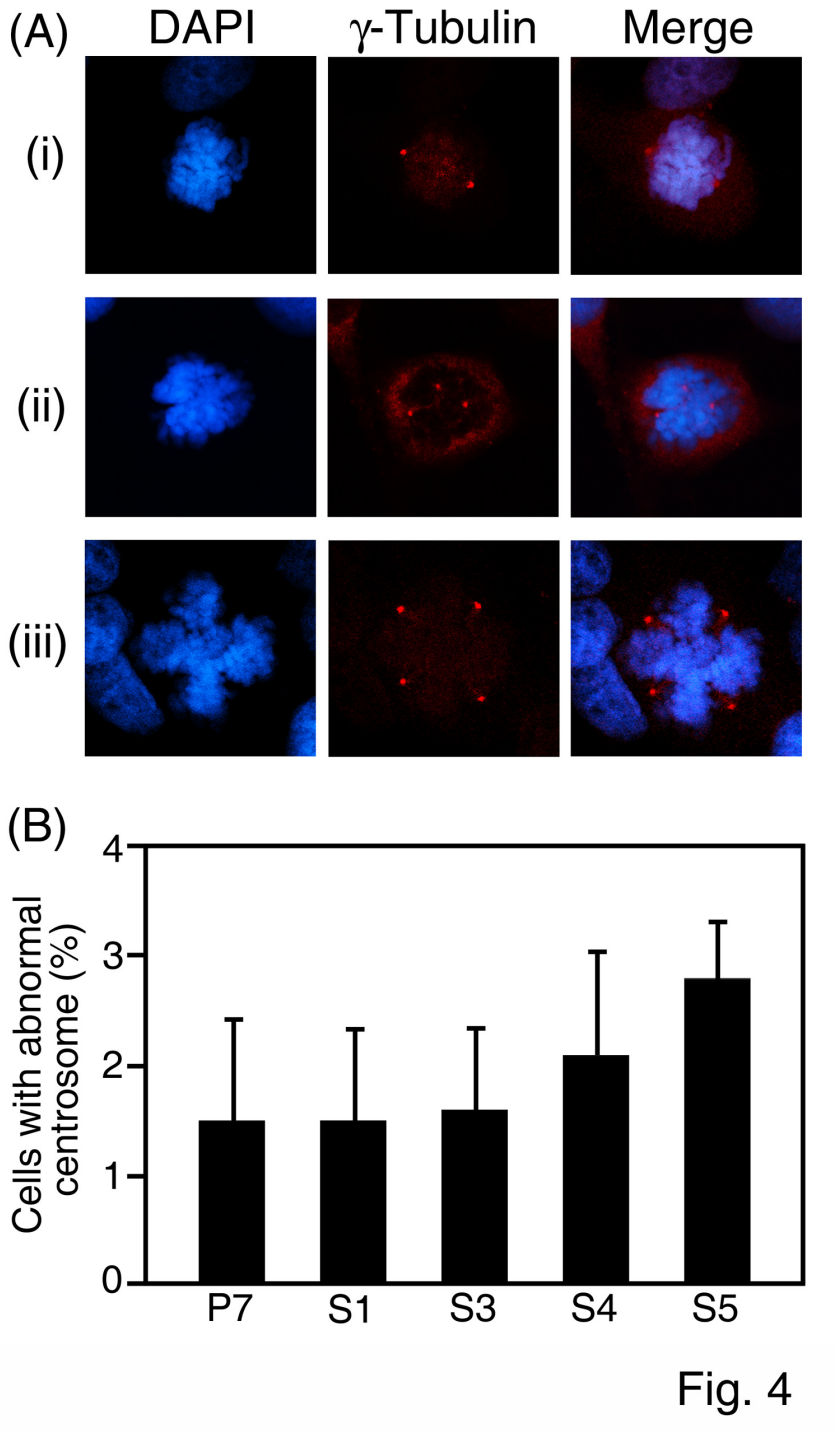
**Representative images of supernumerary centrosomes at the mitotic stage in UL76-expressing cells**. (A) Centrosomes were visualized by staining with γ-tubulin (red) and the chromosomes were visualized by DAPI stain (blue). Normal bipolar centrosome (i) at the mitotic stage is shown. Abnormal centrosome images, tricentrosome (ii) and tetracentrosome (iii) in mitotic cells. Two side-by-side panels of single labeled immunofluorescent images and a third panel with an overlapping image are shown. (B) Quantification of mitotic cells with supernumerary centrosomes in cells stably expressing HCMV UL76 (S1, S3, S4, and S5) and control vector (P7).

Two centrosomes for two spindle poles are present during the mitotic phase of cell division (Fig. 4A(i)) and the presence of more than two centrosomes is considered abnormal (Fig. 4A(ii) and 4A(iii)). The percentage of UL76-expressing cells with more than two centrosomes was marginally increased compared to P7 control cells [S1 (1.5%), S3 (1.6%), S4 (2. 1%), S5 (2.8%), and P7 (1.5%)].

In summary, our data suggest that chromosomal abnormalities, micronuclei, lagging and bridging are significantly induced in UL76-expressing cells. However, centrosome number and spindle network, two key structures that maintain the fidelity of progression through the mitotic phase, did not appear significantly affected. Based on these observations, we investigated whether the cell signals of DNA damage were induced normally.

### The DNA damage signal γ-H2AX is activated in UL76-expressing cells

**Figure 5:**
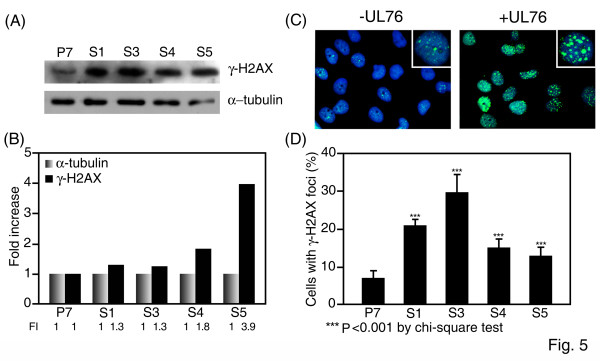
**UL76 induces γ-H2AX foci**. (A) Western blot analysis of γ-H2AX protein levels in cells. α-tubulin was used as a loading control. (B) Quantification of the increase of γ-H2AX protein levels in U-373 MG cells stably expressing UL76 (S1, S3, S4, and S5) after normalization against the α-tubulin loading control and P7 control cells. FI: fold increase. (C) Representative image of γ-H2AX foci (green) in control P7 cells (-UL76) or in UL76-expressing cells (+UL76). Nuclei were counterstained with DAPI (blue). Magnification of the foci images is shown. (D) Quantification of high focal γ-H2AX staining in cells stably-expressing UL76 (S1, S3, S4, and S5) compared with control P7 cells. The average data point was calculated from three independent experiments. At least 1000 cells were counted for each cell line.

Phosphorylation of the histone H2A family members is an initial response to DNA damage. The recruitment of γ-H2AX to the break point develops into visible foci in the nucleus [33]. The level of activated γ-H2AX (Ser139) in UL76-expressing cells was analyzed by Western blotting (Fig. 5A). The relative fold-increase of γ-H2AX (Ser139) in UL76-expressing cells compared to P7 control cells are as follows (Fig. 5B): S1 (1.3), S3 (1.3), S4 (1.8), and S5 (3.9). Consistent with these data, the γ-H2AX foci in the control P7 cells were fine, punctuate, and few, whereas the numbers and sizes of foci were significantly increased in UL76-expressing cells (Fig. 5C). When the γ-H2AX foci were enumerated all UL76-expressing cells that were examined displayed significant increases in the speckled foci (Fig. 5D).

### In vivo expression of UL76 stimulates cellular DNA breaks

**Figure 6:**
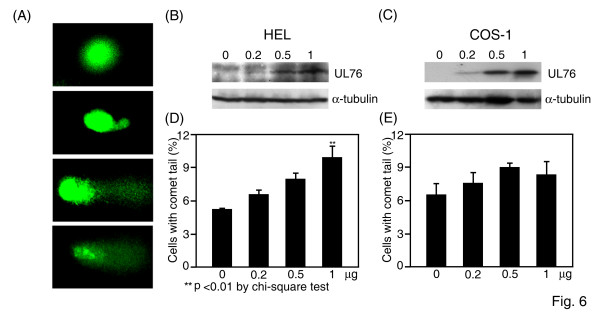
**HCMV UL76 induces DNA breaks in vivo**. (A) Representative images of the nucleus without comet tails (top panel) and nuclei with comet tails (lower three panels). Western blot analysis of UL76 protein levels in HEL299 (B) or COS-1 (C) cells transiently transfected with UL76-expressing plasmid at 0, 0.2, 0.5 and 1.0 μg per 6-cm dish. Cells were lysed in loading buffer, and α-tubulin was used as loading control. Quantification of HEL299 (D) or COS-1 (E) cells with fluorescent comet tails. Each data point was calculated from three independent experiments and is plotted as a percentage of the total cell population.

Comet assays were performed next to investigate whether UL76 was responsible for induced DNA damage. Transfected cells were lysed in an alkaline solution and were subjected to electrophoresis followed by staining for DNA. Undamaged DNA migrated slower and remained within the nucleus (head) (Fig. 6A, top panel), whereas both the single- and double-stranded damaged DNA were observed as tails moving away from the cell during electrophoresis (Fig. 6A, lower three panels). HCMV permissive HEL299 cells and non-permissive COS-1 cells were transiently transfected with 0, 0.2, 0.5, or 1.0 μg of pEF1-UL76 DNA. Western blot analysis demonstrated that levels of UL76 increased with increasing concentrations of DNA in both HEL299 (Fig. 6B) and COS-1 (Fig. 6C) cells. The cells with comet tails were scored and the results are depicted in Fig. 6D and Fig. 6E, respectively. HEL299 cells expressing UL76 produced comet tails that increased in frequency with increasing concentrations of transfected DNA. These increases were statistically significant at concentrations of one μg of transfected DNA. Similar results were obtained when UL76 was expressed in COS-1 cells, with the exception that the percentage of COS-1 cells with comet tails decreased slightly at DNA concentrations higher than one μg.

## Discussion

The HCMV UL76 protein is a member of the highly conserved protein family including herpes simplex virus (HSV-1 and 2) UL24 and murine gammaherpesvirus 68 (MHV68) ORF20. To characterize the function of the UL76 protein family, the initial effort employed an animal model to evaluate infectivity of HSV-1 encoding a defective UL24, which suggested that viruses with mutations in UL24 show decreases in productive replication and reactivation efficiency of latent virus from ganglion [34]. Consistent with these observations, mice and guinea pigs infected with HSV-2 containing the mutated UL24 exhibit less genital lesions compared to that of wild type virus [35]. Both reports are consistent with the characteristics of HCMV UL76, which is involved in viral lytic production and in latency [27,28,30,31].

Second, it is documented that (MHV68) ORF20, a homolog HCMV UL76, induces DNA damage, apoptosis and arrests the cell cycle at the G2/M phase as a result of inactivation of the kinase activity of the cyclin B/cdc2 complex [36,37]. Finally, sequence analyses indicate the family members contain potential endonuclease PD-(D/E)XK motifs [32,38]. This prediction is in agreement with previous speculation that the HCMV UL76 and UL77 proteins, homologs of HSV UL24 and UL25, respectively, may be involved in the final stages of genome cleavage and packaging [12,39,40].

In this report, we present evidence showing that stably transfected cells expressing UL76 accumulate multiple chromosome aberrations. Micronuclei (MN) were first noted in interphase in UL76-expressing cells (Fig. 1). Micronucleus formation is known to derive from incorrectly aligned chromosomes in metaphase (displaced chromosomes), as well as lagging and bridging chromosomes [41]. Consistent with this result and the previous documentation, the ratios of chromosomal misalignments in UL76-expressing mitotic cells, both lagging and bridging, were statistically significant. However, these cells did not appear to trigger responses to DNA damage that are detrimental, suggesting that UL76 may be involved in the evasion of DNA damage responses, cell cycle checkpoint surveillance or inhibition of DNA repair machinery. We investigated signaling pathways that are responsive to DNA breaks in attempts to derive a mechanism for the observed responses. Immediately following DNA damage, histone H2AX is phosphorylated by phosphoinositide 3-kinase like kinases ATM, ATR, and DNA-PKs [42]. Phosphorylated H2AX (γ-H2AX) is recruited to the broken DNA and are visible as foci in cell nuclei. The levels of γ-H2AX and percentage of foci increased in UL76-expressing cells, suggesting the signal for DNA damage is activated and functional. The induction of DNA breaks by UL76 was further evaluated by Comet assay. We observed that cells developed comet tails when UL76 was transiently expressed in both HCMV permissive HEL299 cells and in non-permissive COS-1 cells. This property is in contrast to the response elicited by HSV-1 infection, in which the induction of the DNA damage response occurs only within permissive cells [43]. Moreover, the percentage of cells with comet tails increased as the levels of UL76 increased, suggesting that UL76 plays a role in DNA breakage. Induction of DNA damage may reflect potential activity of UL76 as an endonuclease [32].

In addition to the induction of DNA breaks many viral proteins also affect the spindle network or centrosome number in mitotic cells, which may lead to the emergence of aneuploidy cells [44,45]. The correlation between extra centrosome number and aneuploidy is visibly observed by images that show that supernumerary centrosomes promote the emergence of lagging chromosomes during anaphase. In contrast, cells with supernumerary centrosomes undergoing multipolar cell divisions are almost non-viable [46]. This report may explain the fact that cells with a multiple centrosome number were minimally detected in UL76-expressing cells.

## Conclusion

In summary, our findings suggest that UL76 induces chromosome aberrations. Apparently, cells stably expressing UL76 are not capable of fully repairing the damage because we observed that micronuclei, chromosomal lagging and bridging accumulate in cells constitutively expressing UL76. It is of interest to explore the mechanisms involved in the emergence of chromosome aberrations, including endonuclease activity, evasion of the DNA damage response and mitigation of checkpoint surveillance exerted by HCMV UL76.

## Competing interests

The authors declare that they have no competing interests.

## Authors' contributions

VKS performed the immunofluorescent cell staining, comet assay, Western blotting and statistical analyses. CYD conceived part of this study and provided the stable cell lines. SKW conceived this study, drafted the manuscript, and performed part of the immunofluorescent cell staining.
